# Assessing Public Knowledge and Acceptance of Using Artificial Intelligence Doctors as a Partial Alternative to Human Doctors in Saudi Arabia: A Cross-Sectional Study

**DOI:** 10.7759/cureus.64461

**Published:** 2024-07-13

**Authors:** Atheer Ayed M Alshutayli, Faisal M Asiri, Yazeed Bader Arshi Abutaleb, Bayan Abdullatif Alomair, Abdulelah K Almasaud, Abdullah Almaqhawi

**Affiliations:** 1 College of Medicine, Qassim University, Qassim, SAU; 2 College of Medicine, Prince Sattam Bin Abdulaziz University, Al-Kharj, SAU; 3 College of Medicine, Jazan University, Jazan, SAU; 4 College of Medicine, King Faisal University, Al-Ahsa, SAU; 5 Family Medicine, King Faisal University, Al-Ahsa, SAU

**Keywords:** healthcare, saudi arabia, acceptance, knowledge, artificial intelligence

## Abstract

Objective

To assess the public acceptance of using artificial intelligence (AI) doctors to diagnose and treat patients as a partial alternative to human physicians in Saudi Arabia.

Methodology

An observational cross-sectional study was conducted from January to March 2024. A link to an online questionnaire was distributed through social media applications to citizens and residents aged 18 years and older across various regions in Saudi Arabia. The sample size was calculated using the Raosoft online survey size calculator, which estimated that the minimum sample size should be 385.

Results

Of the 386 participants surveyed, 85.8% reported being aware of AI, and 47.9% reported having some knowledge about different AI fields in daily life. However, almost one-third (32.9%) reported a lack of knowledge about the use of AI in healthcare. In terms of acceptance, 52.3% of respondents indicated they felt comfortable with the use of AI tools as partial alternatives to human doctors, and 30.8% believed AI is useful in the field of health. The most common concern (63.7%) about the use of AI tools accessible to patients was the difficulty of describing symptoms using these tools.

Conclusion

The findings of this study provide valuable insights into the public’s knowledge and acceptance of AI in medicine within the Saudi Arabian context. Overall, this study underscores the importance of proactively addressing the public's concerns and knowledge gaps regarding AI in healthcare. By fostering greater understanding and acceptance, healthcare stakeholders can better harness the potential of AI to improve patient outcomes and enhance the efficiency of medical services in Saudi Arabia.

## Introduction

Artificial intelligence (AI) is the science and engineering of creating intelligent machines using algorithms or rules to simulate human intellectual functions, such as learning and problem-solving [1]. AI has demonstrated significant growth and development in recent years, especially following the development of large language models, such as Chat Generative Pre-trained Transformer (ChatGPT), which are available for public use [2]. This growth is reflected in the medical field through multiple applications of AI in various specialties, such as radiology, dermatology, pathology, and ophthalmology [3-6].

Research assessing the potential and capabilities of AI has demonstrated equal or even greater potential than physicians in some fields, such as radiology [2,7-10]. AI shows great potential to revolutionize healthcare, offering benefits for both patients and healthcare professionals. One key advantage of AI is improved accuracy and efficacy in medical imaging, especially for early diagnosis and prevention of diseases. This advancement not only improves patient outcomes but also reduces the time and cost of medical scans. AI can also minimize administrative costs by automatically extracting data from therapy notes and valuable information from previous medical records. Other advantages include enhancing patient interaction, employing virtual care, and analyzing data for research [1,11].

Despite these advantages, ethical considerations remain a constant concern. For instance, the increasing demand for data to train AI algorithms in healthcare raises the risk of unauthorized access to sensitive information. Another concern is AI bias, where training AI on a biased dataset could lead to inaccurate outcomes [12]. While healthcare professionals demonstrate increasing knowledge and acceptance of the application of AI in medical practice, public opinion on this integration remains largely unexplored [13-15]. Systematic reviews indicate a general willingness to accept AI applications in healthcare, with data privacy and AI decision-making emerging as key concerns [16,17]. Similar research on public awareness of AI in healthcare conducted in China in 2020 showed that while most individuals had optimistic views and thought AI doctors would eventually replace human ones, either totally or in part, some still harbored skepticism primarily due to a lack of trust in the technology and the absence of the humanistic care component [18]. Systematic reviews revealed that people are aware of the special benefits and practicality of medical AI, yet numerous concerns regarding the use of AI in medicine have been noted, most of which relate to ethical and legal matters [19]. The general perspective of the American public is one of mistrust and discomfort with medical AI. Despite that, there is optimism for the future and a belief that medical care will significantly improve over the next few decades [20]. Other concerns include the potential for AI to provide inaccurate or false information, the inability to build long-term patient-doctor relationships, and to provide ongoing care, which are essential for efficient healthcare management and may pose challenges for AI doctors in matching the empathic and emotional support provided by humans. Additionally, the high cost of AI may pose financial challenges for healthcare institutions [21]. However, research on public perceptions of AI in healthcare, specifically within Saudi Arabia, remains inadequate. This study addressed this gap by employing a survey-based cross-sectional design to assess the public acceptance of using AI doctors to diagnose and treat patients as a partial alternative to human physicians in Saudi Arabia.

## Materials and methods

An observational cross-sectional study was conducted from January to March 2024. A link to an online questionnaire was distributed through social media applications (WhatsApp, Twitter, and Telegram) with an invitation for adult citizens and residents to share their views on AI. Using an estimated average population of Saudi Arabia in 2024 (33,700,000), the sample size was calculated using the Raosoft online survey size calculator at a 95% confidence level, an estimated 50% response distribution, and a margin of error of ±5%. The minimum sample size for this study was calculated as 385.

Participants were recruited using a convenience sampling technique and were requested to sign the informed consent. Data were obtained from all participants via an online questionnaire. The inclusion criteria included Saudi citizens and non-Saudi residents aged at least 18 years who were willing to participate. Those who declined to participate or did not fully complete the questionnaire were excluded. The study goals were presented to all participants at the beginning of the survey, at which time they provided informed consent before continuing. Participants were also informed that all data would be kept confidential and used only for research purposes.

We created the questionnaire based on two studies published in the literature that met our study objectives [22,23]. The survey was written in Arabic and consisted of three sections: demographic information, general knowledge of AI, and acceptance of using AI doctors as partial alternatives to human physicians. A panel of three consultants in family medicine evaluated the validity of the questionnaire, and all suggested changes were applied. A pilot study was conducted with about 10% of the sample size (30 participants) of the target population to evaluate the data collection tools, the respondents' reactions, the sampling technique, and the proposed work plan. Those who participated in the pilot study were excluded from the study population. Ethical approval for this study was obtained from the King Faisal University Research Ethics Committee (KFU-REC-2024-FEB-ETHICS 2002).

SPSS (IBM version 26) was used to complete the statistical analysis. Categorical data were presented as frequencies and percentages. The questions about knowledge and acceptance of AI were summed, resulting in numerical variables, which were categorized. The knowledge categories were (0-4) for poor knowledge, (5-7) for intermediate knowledge, and (8-12) for good knowledge. The acceptance score was calculated, and the analysis was conducted after excluding the participants who answered that they were not aware of the term AI. The acceptance categories were as follows: (0-12) for not accepted, (13-23) for intermediate acceptance, and (24-36) for accepted. The Chi-square test was used to assess the association between knowledge and acceptance of AI and sociodemographic data. Binary logistic regression was conducted to predict the knowledge and acceptance of AI based on sociodemographic data. Results of the regression are presented as odds ratios and their respective 95% confidence intervals. A p-value of < 0.05 was used as an indication of statistical significance.

## Results

The study surveyed 386 participants from Saudi Arabia to assess their perspectives on AI doctors in healthcare. The majority were young adults, with 48.40% aged 18-24 years (Table 1). Most participants were Saudi nationals (97.20%), residing primarily in the central region (43.30%). About 58.80% of the participants were single, and 42.20% held a bachelor's degree. Students comprised the largest occupational group at 42.70%. Furthermore, 70.20% of the participants demonstrated good knowledge of AI. In terms of acceptance, 52.30% expressed openness to AI doctors as partial alternatives to human physicians for diagnosis and treatment.

**Table 1:** Sociodemographic data of participants (N = 386). AI: Artificial intelligence.

Parameters	Category	n	%
Gender	Male	167	43.30%
	Female	219	56.70%
Age	18–24	187	48.40%
	25–34	72	18.70%
	35–44	52	13.50%
	45–54	51	13.20%
	55–64	21	5.40%
	65–74	3	0.80%
Nationality	Saudi	375	97.20%
	Non-Saudi	11	2.80%
Which region of Saudi Arabia do you currently live in?	Northern	5	1.30%
	Southern	106	27.50%
	Central	167	43.30%
	Eastern	83	21.50%
	Western	25	6.50%
Marital status	Single	227	58.80%
	Married	155	40.20%
	Divorced	4	1.00%
Educational level	Elementary	1	0.30%
	Middle	9	2.30%
	Secondary	158	40.90%
	Diploma	34	8.80%
	Bachelor's degree	163	42.20%
	Master's degree and above	21	5.40%
Occupation	Student	165	42.70%
	Unemployed	61	15.80%
	Government employee	95	24.60%
	Private sector employee	37	9.60%
	Free business	7	1.80%
	Retired	21	5.40%
Is your study or work related to the medical field?	No	231	59.80%
	Yes	155	40.20%
Knowledge about AI	Poor knowledge	43	11.10%
	Intermediate knowledge	72	18.70%
	Good knowledge	271	70.20%
Acceptance of AI	Not accepted	20	6.00%
	Intermediate acceptance	138	41.70%
	Accepted	173	52.30%

**Figure 1:**
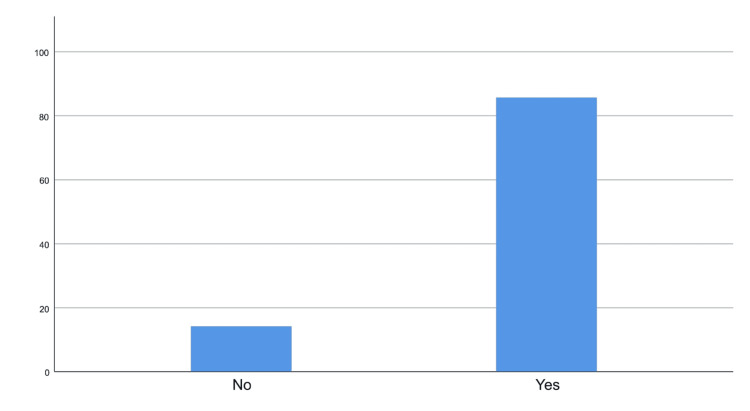
Bar chart showing the awareness levels of AI. AI: Artificial intelligence.

**Figure 2:**
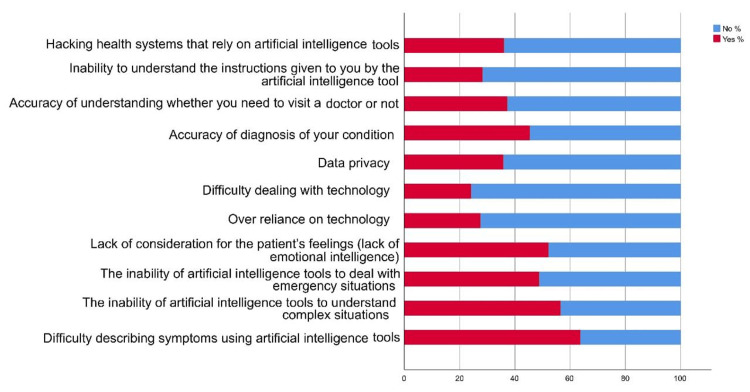
Reported concerns about the use of artificial intelligence tools.

**Figure 3:**
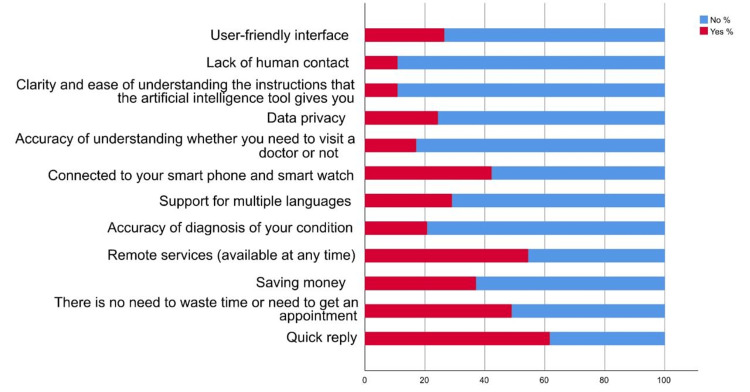
Reported advantages of using artificial intelligence tools.

The survey reveals a high level of awareness (85.80%) among participants regarding AI (Figure 1), with almost half (47.90%) reporting some knowledge of its applications in daily life (Table 2). In terms of healthcare, a quarter (25.40%) of respondents have knowledge of AI use. Encounters with AI tools are common, with nearly 40% having encountered 2-4 such tools (Figure 2). The perceived usefulness of AI is notable, as about 28.50% find it very useful in their work or daily life, and 30.80% consider it useful in healthcare (Figure 3). Primary concerns include the difficulty in articulating symptoms through AI tools (63.70%), particularly among younger adults, females, and students. Despite concerns, quick replies from AI tools (61.70%) are seen as the most encouraging factor for their use, especially among similar demographic groups.

**Table 2:** Participants' knowledge about artificial intelligence (N = 386).

Parameters	Category	n	%
Have you ever encountered the term artificial intelligence?	No	55	14.20%
	Yes	331	85.80%
Do you know the different fields in which artificial intelligence is used in your daily life?	No	44	11.40%
	To some extent	185	47.90%
	Yes	157	40.70%
Do you know how artificial intelligence is used in the field of health?	No	127	32.90%
	To some extent	161	41.70%
	Yes	98	25.40%
How many artificial intelligence tools have you encountered in your life?	None	107	27.70%
	1	74	19.20%
	2–4	154	39.90%
	More than 4	51	13.20%
How useful is artificial intelligence in your field of work or daily life?	I have no knowledge of artificial intelligence	15	3.90%
	I have never used it	84	21.80%
	Limited use	69	17.90%
	Useful	108	28.00%
	Very useful	110	28.50%
How useful is artificial intelligence in the field of health?	I have no knowledge of artificial intelligence	26	6.70%
	I have never used it	88	22.80%
	Limited use	40	10.40%
	Useful	119	30.80%
	Very useful	113	29.30%

As reported in Table 3, 47.90% of participants believe AI tools will not replace the need to visit a doctor, 59.60% disagree that AI will replace clinical examinations, and 68.70% disagree that AI will replace laboratory testing. Notably, 50.50% expressed uncertainty about the ability of AI tools to provide solutions matching health and financial capabilities, and 50.50% expressed uncertainty about the ability of AI tools to ask the necessary questions about medical history. Regarding the replacement of doctors by AI, 43.80% disagree that AI will replace human physicians now or in the future.

**Table 3:** Participants' perspectives about the use of an artificial intelligence doctor as a partial alternative for a human physician (N = 386).

Parameters	Category	n	%
Do you think that AI tools will replace the need to go to the doctor?	No	185	47.90%
	Sometimes	177	45.90%
	Yes	24	6.20%
Do you think that artificial intelligence tools are able to ask you the necessary questions about your medical history, or will it ask you about everything?	No	74	19.20%
	Sometimes	195	50.50%
	Yes	117	30.30%
Do you think that artificial intelligence tools will replace the need for clinical examination?	No	230	59.60%
	Sometimes	114	29.50%
	Yes	42	10.90%
Do you think that artificial intelligence tools will replace laboratory testing?	No	265	68.70%
	Sometimes	88	22.80%
	Yes	33	8.50%
If artificial intelligence could diagnose you, do you think that artificial intelligence tools would give you a solution that matches your health and financial capabilities?	No	92	23.80%
	Maybe	195	50.50%
	Yes	99	25.60%
Will artificial intelligence replace human doctors?	No	169	43.80%
	Probably	81	21.00%
	Yes, within this year	3	0.80%
	In the distant future	83	21.50%
	I don't know	50	13.00%

The analysis of respondents' knowledge about AI revealed significant variations across demographic factors (Table 4). Age was a significant determinant (P < 0.001), with the highest knowledge levels observed among those aged 18-24 years (82.40%). Marital status also played a significant role (P < 0.001), with singles showing the highest knowledge levels (80.20%). Educational attainment was significant (P = 0.022), with higher education correlating with better AI knowledge. Occupation had a profound impact (P < 0.001), with students having the highest knowledge levels (86.10%) compared to other groups. Logistic regression confirmed these associations, showing significantly lower odds of good knowledge among unemployed individuals (OR = 0.026, 95% CI: 0.004-0.162) and various other occupational categories compared to students (Table 5). These findings underscore the influence of age, marital status, education, and occupation on AI knowledge levels among respondents.

**Table 4:** Knowledge of artificial intelligence based on sociodemographic characteristics. * Chi-square test.

Parameter	Category	Poor knowledge		Intermediate knowledge		Good knowledge		P-value*
		N	%	N	%	N	%	
Gender	Male	18	10.80%	33	19.80%	116	69.50%	0.882
	Female	25	11.40%	39	17.80%	155	70.80%	
Age	18–24	7	3.70%	26	13.90%	154	82.40%	< 0.001
	25–34	10	13.90%	14	19.40%	48	66.70%	
	35–44	9	17.30%	12	23.10%	31	59.60%	
	45–54	8	15.70%	13	25.50%	30	58.80%	
	55–64	7	33.30%	7	33.30%	7	33.30%	
	65–74	2	66.70%	0	0.00%	1	33.30%	
Nationality	Saudi	43	11.50%	70	18.70%	262	69.90%	0.477
	Non-Saudi	0	0.00%	2	18.20%	9	81.80%	
Which regions of Saudi Arabia do you currently live in?	Northern	0	0.00%	2	40.00%	3	60.00%	0.054
	Southern	10	9.40%	19	17.90%	77	72.60%	
	Central	12	7.20%	28	16.80%	127	76.00%	
	Eastern	16	19.30%	19	22.90%	48	57.80%	
	Western	5	20.00%	4	16.00%	16	64.00%	
Marital status	Single	11	4.80%	34	15.00%	182	80.20%	< 0.001
	Married	32	20.60%	37	23.90%	86	55.50%	
	Divorced	0	0.00%	1	25.00%	3	75.00%	
Educational level	Elementary	0	0.00%	0	0.00%	1	100.00%	0.022
	Middle	3	33.30%	2	22.20%	4	44.40%	
	Secondary	15	9.50%	26	16.50%	117	74.10%	
	Diploma	9	26.50%	8	23.50%	17	50.00%	
	Bachelor's degree	13	8.00%	35	21.50%	115	70.60%	
	Master's degree and above	3	14.30%	1	4.80%	17	81.00%	
Occupation	Student	2	1.20%	21	12.70%	142	86.10%	< 0.001
	Unemployed	14	23.00%	13	21.30%	34	55.70%	
	Government employee	14	14.70%	20	21.10%	61	64.20%	
	Private sector employee	6	16.20%	9	24.30%	22	59.50%	
	Free business	0	0.00%	2	28.60%	5	71.40%	
	Retired	7	33.30%	7	33.30%	7	33.30%	

**Table 5:** Association between age, marital status, and occupation and knowledge about artificial intelligence. * Binary logistic regression analysis.

Parameters	Category	OR	95% CI		P-value*
			LB	UB	
Age	18–24	Ref.	Ref.	Ref.	Ref.
	25–34	1.130	0.239	5.355	0.878
	35–44	1.202	0.181	8.001	0.849
	45–54	1.658	0.225	12.201	0.619
	55–64	0.846	0.090	7.928	0.883
	65–74	0.143	0.004	5.018	0.284
Marital status	Single	Ref.	Ref.	Ref.	Ref.
	Married	0.402	0.098	1.647	0.205
Occupation	Student	Ref.	Ref.	Ref.	Ref.
	Unemployed	0.026	0.004	0.162	< 0.001
	Government employee	0.038	0.005	0.293	0.002
	Private sector employee	0.052	0.007	0.407	0.005
	Retired	0.069	0.006	0.785	0.031

Participants in the study generally hold a positive view of AI tools in healthcare, with a significant proportion (44.70%) acknowledging their potential to determine the necessity of visiting a doctor (Table 6). A majority also believe AI will enhance healthcare (47.70%) and improve public health (42.60%). About 37.80% of respondents agree that the benefits of AI tools outweigh the negatives, and 40.50% see AI as capable of reducing health risks due to delayed medical care. Notably, 40.80% of participants think AI accessible to patients could save time. However, opinions varied on whether to accept AI diagnoses, with some expressing skepticism compared to diagnoses made by primary care doctors. Despite some skepticism, 40.20% perceive AI tools as potentially reducing medical fatigue among doctors.

**Table 6:** Acceptance of artificial intelligence (N = 331, after excluding participants who were not aware of artificial intelligence).

Parameters	Category	n	%
Do you think that incorporating artificial intelligence tools as health assistants that are accessible and accessible to patients before visiting a doctor can help determine the need for a human doctor or not?	Strongly disagree	13	3.90%
	Disagree	30	9.10%
	Neutral	93	28.10%
	Agree	148	44.70%
	Strongly agree	47	14.20%
Do you think that the use of artificial intelligence tools that are easily accessible by patients will enhance health care in Saudi Arabia?	Strongly disagree	0	0.00%
	Disagree	18	5.40%
	Neutral	67	20.20%
	Agree	158	47.70%
	Strongly agree	88	26.60%
Do you believe that the use of artificial intelligence tools that are easily accessible by patients will improve public health?	Strongly disagree	0	0.00%
	Disagree	27	8.20%
	Neutral	78	23.60%
	Agree	141	42.60%
	Strongly agree	85	25.70%
Do you think that the benefits of using artificial intelligence tools that are easily accessible by patients will outweigh the negatives?	Strongly disagree	16	4.80%
	Disagree	26	7.90%
	Neutral	110	33.20%
	Agree	125	37.80%
	Strongly agree	54	16.30%
Do you think that adopting artificial intelligence tools that are easily accessible by patients will reduce the health risks resulting from delayed access to medical care?	Strongly disagree	9	2.70%
	Disagree	23	6.90%
	Neutral	77	23.30%
	Agree	134	40.50%
	Strongly agree	88	26.60%
AI tools that are easily accessible to patients have the potential to save time.	Strongly disagree	4	1.20%
	Disagree	17	5.10%
	Neutral	50	15.10%
	Agree	135	40.80%
	Strongly agree	125	37.80%
Would you accept a diagnosis from medical artificial intelligence tools?	Strongly disagree	54	16.30%
	Disagree	56	16.90%
	Neutral	68	20.50%
	Agree	107	32.30%
	Strongly agree	46	13.90%
Would you trust artificial intelligence diagnoses more than those made by primary care doctors?	Strongly disagree	86	26.00%
	Disagree	68	20.50%
	Neutral	72	21.80%
	Agree	78	23.60%
	Strongly agree	27	8.20%
Do you think that relying on artificial intelligence tools that are easily accessible by patients may reduce medical fatigue on doctors?	Strongly disagree	11	3.30%
	Disagree	16	4.80%
	Neutral	60	18.10%
	Agree	133	40.20%
	Strongly agree	111	33.50%

The analysis of factors influencing the acceptance levels of AI among respondents revealed several significant associations (Table 7). Age exhibited a significant association with acceptance levels (P = 0.035), with a higher proportion of individuals aged 35-44 years (75.00%) reporting acceptance compared to other groups. Occupation also showed a significant association (P = 0.045), with higher acceptance observed among government employees (64.50%). However, gender, nationality, region of residence, marital status, and educational level did not exhibit significant associations with acceptance levels of AI.

**Table 7:** Acceptance of artificial intelligence based on sociodemographic characteristics. * Chi-square test.

Parameters	Category	Not accepted		Intermediately accepted		Accepted		P-value*
		N	%	N	%	N	%	
Gender	Male	9	6.60%	66	48.20%	62	45.30%	0.097
	Female	11	5.70%	72	37.10%	111	57.20%	
Age	18–24	10	5.70%	84	48.00%	81	46.30%	0.035
	25–34	4	6.70%	21	35.00%	35	58.30%	
	35–44	3	7.50%	7	17.50%	30	75.00%	
	45–54	1	2.40%	18	43.90%	22	53.70%	
	55–64	2	14.30%	8	57.10%	4	28.60%	
	65–74	0	0.00%	0	0.00%	1	100.00%	
Nationality	Saudi	20	6.20%	135	41.70%	169	52.20%	0.792
	Non-Saudi	0	0.00%	3	42.90%	4	57.10%	
Which regions of Saudi Arabia do you currently live in?	Northern	0	0.00%	1	25.00%	3	75.00%	0.299
	Southern	3	3.20%	35	37.20%	56	58.60%	
	Central	12	8.40%	59	41.30%	72	50.30%	
	Eastern	2	3.00%	31	47.00%	33	50.00%	
	Western	3	12.50%	12	50.00%	9	37.50%	
Marital status	Single	13	6.20%	96	45.90%	100	47.80%	0.075
	Married	6	5.10%	42	35.60%	70	59.30%	
	Divorced	1	25.00%	0	0.00%	3	75.00%	
Educational level	Elementary	0	0.00%	1	100.00%	0	0.00%	0.209
	Middle	1	14.30%	2	28.60%	4	57.10%	
	Secondary	8	5.80%	65	47.40%	64	46.70%	
	Diploma	1	4.80%	5	23.80%	15	71.40%	
	Bachelor's degree	9	6.20%	62	42.50%	75	51.40%	
	Master's degree and above	1	5.30%	3	15.80%	15	78.90%	
Occupation	Student	8	5.20%	77	49.70%	70	45.20%	0.045
	Unemployed	5	9.80%	14	27.50%	32	62.70%	
	Government employee	2	2.50%	25	32.90%	49	64.50%	
	Private sector employee	4	12.90%	12	38.70%	15	48.40%	
	Free business	0	0.00%	3	50.00%	3	50.00%	
	Retired	1	8.30%	7	58.30%	4	33.30%	

The analysis did not find a significant association between knowledge levels about AI and acceptance levels among respondents (P = 0.143) (Table 8). Among those with poor knowledge of AI, 60.00% reported intermediate acceptance and 35.00% reported acceptance. Similarly, respondents with intermediate knowledge showed 45.60% with intermediate acceptance and 43.90% with acceptance. Conversely, among respondents with good knowledge of AI, a majority (55.50%) reported acceptance, with only 5.10% indicating a lack of acceptance. These results suggest a trend where higher knowledge levels about AI might correlate with greater acceptance, emphasizing the potential role of education in fostering acceptance and utilization of AI technologies in healthcare.

**Table 8:** Association between knowledge and acceptance of artificial intelligence. * Chi-square test.

Parameters	Category	Not accepted		Intermediate acceptance		Accepted		P-value*
		N	%	N	%	N	%	
Knowledge about artificial intelligence	Poor knowledge	1	5.00%	12	60.00%	7	35.00%	0.143
	Intermediate knowledge	6	10.50%	26	45.60%	25	43.90%	
	Good knowledge	13	5.10%	100	39.40%	141	55.50%	

## Discussion

This study advances the understanding of the acceptance of AI. The results offer four significant insights: First, while a majority of the participants (85.8%) are aware of AI, only 30% recognize its usefulness in healthcare. Second, quick responses were noted as the most encouraging factor for using AI tools, although the difficulty in describing symptoms using AI tools was the most common concern. Third, nearly half of the participants believed AI tools would not replace the need to visit a doctor. Fourth, the results indicate a generally positive attitude toward AI tools as health assistants.

Research exploring the intersection of AI acceptance and knowledge in healthcare is still limited. Although no studies directly addressing our specific research question were identified, existing literature provides relevant points for discussion.

In this study, 52.3% of participants accept AI doctors as a partial alternative to human doctors. This compares favorably with findings by Tamori H et al. [23], who reported 55.2% of participants had a good knowledge of AI. However, only 30% of our participants were aware of AI's usefulness in healthcare, contrasting with Tamori H et al.'s findings that participants had confidence in AI’s usefulness and its future necessity in medicine.

Educational level was significantly associated with acceptance, a finding similar to that of Tamori H et al. [23], who noted that experts were more receptive to using AI compared to the general public. In our study, the highest proportion of good knowledge was observed in the 18-24 age group (82.40%), and a significant majority were students. Furthermore, a study focused on medical students [24] found that they were confident in AI's ability to assist in diagnosis, reflecting a greater familiarity with technologies like AI among younger generations. Additionally, a large percentage of our sample with bachelor's degrees (70.60%) showed good knowledge, suggesting a correlation between higher education levels and greater knowledge of AI. Digital inequality plays a crucial role in understanding AI knowledge levels in healthcare. Our study revealed higher levels of AI knowledge among younger and more educated participants. However, a significant portion of the population with limited access to digital technologies may not benefit equally from AI advancements [25]. Acceptance levels of AI were highest among those with good knowledge (55.70%), indicating a strong correlation between understanding and acceptance. However, most people do not believe AI will replace the need for clinical examinations and doctor visits, which suggests a high level of trust in human doctors. Additionally, about half of the participants believe AI will eventually replace doctors in clinical settings. Despite this, the survey covered a diverse range of occupational backgrounds, underscoring AI's importance across professional fields. Gender, region of residence, and marital status did not show significant associations with acceptance levels.

Regarding the replacement of doctors by AI, 43.8% of our participants disagreed. However, a previous study focusing on a more specific area found that 41% of respondents in Germany favored using AI alone to diagnose melanoma [26]. In Sweden, 38% of participants in a breast cancer screening program preferred computer-only reading [9]. Furthermore, 35.4% of Korean doctors agreed that AI could replace them in their jobs [27]. Meanwhile, concerns about the costs of using AI were similar to those found by Tamori H et al. [23]. More than 60% of our participants did not view cost savings as an encouraging factor. Also, Tamori H et al. [23] demonstrate that their participants think AI will increase costs. This is probably due to the free healthcare services provided to the Saudi community, unlike in Japan. Data privacy was a strong concern reported by our participants. Similarly, Tamori H et al. found that about half of the respondents expressed moderate or strong concerns about data leakage [23].

In terms of disadvantages, more than half of the participants believe it is hard to use AI to express symptoms, which raises concerns about its usefulness in medical settings. This indicates that patients should be educated on using AI in the medical profession, and clear instructions are needed when using AI tools to ensure optimal use, especially for older individuals. The attitude toward AI tools as health assistants showed that 44.70% of participants agreed these tools can help determine the need for a human doctor.

Regarding the balance between benefits and negatives, 37.80% agreed that the benefits would outweigh the negatives in terms of reducing health risks resulting from delayed access to medical care, and AI tools accessible to patients could save time. This may be due to society in Saudi Arabia being satisfied with the usual medical service provided by health practitioners, which stimulates their lack of interest in new technology and its advantages, and their fear of changing to something new that they are not accustomed to. However, opinions were more divided concerning acceptance and trust in AI diagnoses, with 32.30% willing to accept a diagnosis from medical AI tools, and 26.00% disagreeing that they would trust AI diagnoses more than those made by primary care doctors. Nonetheless, 40.20% of our population believed that reliance on accessible AI tools could reduce medical fatigue among doctors.

Limitations

The data collected from the participants consisted of their self-reported responses, and their knowledge levels influenced their attitudes and acceptance of AI. Since the responses are based on opinions, feelings, and preferences, this increases the risk of response bias. Also, the reliance on social media platforms for participant recruitment may have introduced selection bias by excluding people who do not use these platforms frequently, potentially resulting in an overestimation of overall acceptance for AI doctors. The current study's finding that 48.40% of participants were aged 18-24, with participation decreasing in older age groups, highlights the potential for underrepresentation of older adults who may have different attitudes and experiences with technology. Additionally, the survey was conducted in Saudi Arabia, where cultural norms and ethics may affect participants' attitudes towards being diagnosed with modern technology versus a human physician, especially among older adults. Other limitations include unmeasured variables such as media exposure, individual attitudes, or social networks that affect participants' awareness and acceptance of AI in healthcare.

To gain a more comprehensive understanding of different perspectives on AI in healthcare, future research should employ recruitment strategies that reach beyond social media users. Ideally, random or stratified sampling techniques should be used to ensure a representative sample of the population. Additionally, including a more geographically diverse sample would provide valuable insights into how cultural contexts influence acceptance of AI in medicine. Furthermore, longitudinal studies should be conducted to compare attitudes and acceptance of AI in the medical field over time. Healthcare providers and AI developers should collaborate to understand the attitudes and behaviors of patients and how they can provide a better experience through the use of AI and improve patients' quality of life.

## Conclusions

The findings of this study provide valuable insights into the public's knowledge and acceptance of AI in medicine within the Saudi Arabian context. Overall, this study emphasizes the importance of proactively addressing the public's concerns and knowledge gaps regarding AI in healthcare. By fostering greater understanding and acceptance, healthcare stakeholders can better utilize the potential of AI to improve patient outcomes and enhance the efficiency of medical services in Saudi Arabia.
